# Motivators and barriers to research participation among medical students in Saudi Arabia

**DOI:** 10.1371/journal.pone.0284990

**Published:** 2023-04-27

**Authors:** Rakan K. Alhabib, Noara Alhusseini, Anas G. Aboalsamh, Ghaith Adi, Aya Ismail, Amro Hajja, Duaa Alammari, Ziad Khalil, Maha A. Alharbi, Sarah K. Albahiti

**Affiliations:** 1 College of Medicine, Alfaisal University, Riyadh, Saudi Arabia; 2 Department of Biostatistics, Epidemiology and Public Health, College of medicine, Alfaisal University, Riyadh, Saudi Arabia; 3 College of Medicine, King Abdulaziz University, Jeddah, Saudi Arabia; 4 King Abdullah International Medical Research Center, College of Medicine, King Saud bin Abdulaziz University for Health Sciences (KSAU-HS), Riyadh, Saudi Arabia; 5 King Saud bin Abdulaziz University for Health Sciences (KSAU-HS), Riyadh, Saudi Arabia; 6 Radiology Department, College of Medicine, Kind Abdulaziz University, Riyadh, Saudi Arabia; BRAC University, BANGLADESH

## Abstract

Little is known about the obstacles medical students face when conducting research in Saudi Arabia. Moreover, the proportion of medical students in research has been unknown in our region compared to other regions. We sought to identify the barriers and motivators that influence undergraduate medical students in pursuing research. This was a cross-sectional study design, utilizing an online survey distributed through social media platforms from the 17^th^ of December 2021 to the 8^th^ of April 2022. The survey was distributed to four universities in Saudi Arabia. Participants’ characteristics, details regarding involvement in research, and attitude towards research were collected. Frequency measures were used to characterize the demographics and chi-squared tests to determine associations. A total of 435 students were included in the final analysis. The highest proportion of students that responded were second year, followed by first year medical students. Less than half (47.6%) of medical students were involved in research. A significant correlation was revealed between the involvement in research and higher participants’ Grade Point Average (GPA). The top three incentives for pursuing undergraduate research were “admission into residency programs” (44.8%), “interest in research” (28.7%), and “financial return” (10.8%). However, the top three limitations were “lack of time” (29.2%), “lack of mentoring” (16.8%), and “lack of interest in research” (14.7%). System-related barriers and motivators were the main reasons behind the involvement of medical students in research. Our study is a call for action to raise awareness among medical students about the importance of research and to provide solutions to overcome these barriers.

## Introduction

Research is the corner stone in both scientific and medical advancement [1]. Playing an essential role in medical education because it clarifies changing patterns of disease and expands knowledge [2]. Exposing medical students to research during the early stages of education can enhance and progress their future careers [3].Research not only educates us but also inspires readers to explore and decide on future educational or professional endeavors. Involving medical students in research activities increases their interest while also helping them understand what they want to pursue with their medical careers. Students who published articles as undergraduates were three times more likely to publish after graduation when compared to their counterparts that were not involved in research [2]. Another advantage of undergraduate research is that it instills critical skills and habits, such as the ability to question, explore, examine, understand, and analyze a plethora of cases. These attributes are vital in the development of critical thinking and creating a good foundation for becoming competent physicians [4]. Many factors may deter students from perusing research including the perceived competitiveness and the heavy demands by their medical education. Another main concern is that students may be considered as less significant, having no input in the research design or critical thinking during the research process [5]. Given the difficulties and competing interests in developing an undergraduate medical curriculum, as well as the outcomes of other learners’ attitudes during medical school, it is critical to investigate the research experience of medical students [6]. The aim of our study is to explore the obstacles and motivators undergraduate medical students face when pursuing research.

## Materials and methods

This was a cross-sectional study design, utilizing an online survey distributed through social media platforms from the 17^th^ of December 2021 to the 8^th^ of April 2022. A structured survey was developed using Microsoft forms. The distributed questionnaire was developed based on assessing various previously published questionnaires [5,7]. All questions were multiple choice questions to ensure proper capture of the data. It was distributed to 4 universities in Saudi Arabia: Alfaisal University, King Abdulaziz University, and King Saud bin AbdulAziz University for Health Sciences (KSAU-HS) in Jeddah and Riyadh. The survey language was English. Participants’ characteristics, details about involvement in research, and the attitude towards research were collected. Due to variable Grade Point Average (GPA) scales between the Saudi universities that were included in the study, GPAs were unified under a 5.0 GPA scale. Participants were asked to give details about the obstacles they faced when perusing research. They were also asked about their involvement in research and the extent to which these variables represent their research experience.

### Primary objective

Identify the barriers and motivators that influence undergraduate medical students in pursuing research.

### Secondary objectives

1, Identify the number of medical students involved in research. 2, Assess associations between the involvement in research and participants’ characteristics (GPA. nationality, and current academic years).

### Inclusion criteria

Undergraduate medical students were within the first to fifth year of medicine at Alfaisal University, King Abdulaziz University, or King Saud bin AbdulAziz University for Health Sciences (KSAU-HS) in Jeddah and Riyadh.

### Exclusion criteria

Students who were not enrolled in classes at the time and dropouts.

### Sample size

The estimated number of students in the four universities is approximately 3000. A random sample was calculated and revealed to be at least 384.

### Statistical analysis

We used frequency measures to characterize the demographics. Chi-squared test was used to determine associations. We used a 95% confidence interval and 5% margin of error with the level of significance set at a P-value <0.05.

### Ethical considerations

The study was reviewed and approved by the IRB (institutional review board) of Alfaisal University in Riyadh (IRBC/1915/21/), King Saud bin Abdulaziz University for Health Sciences in Riyadh and Jeddah (NRC21R/297/07). However, it was exempted in King Abdulaziz University in Jeddah, Saudi Arabia. Participation was voluntary and the names of the respondents were kept anonymous to ensure confidentiality and privacy. All information was kept available only to the authors and has only been used for research purposes.

## Results

The survey obtained a sample size of 435; most students were from Alfaisal University (n = 174, 40%), followed by King Abdulaziz University (n = 93, 21.4%), KSAU-HS in Riyadh (n = 89, 20.5%), and KSAU-HS in Jeddah (n = 79, 18.2%). The sample included slightly more males (56.6%) than females (43.4%). The survey was distributed to the four universities via various social media platforms. Junior students had a higher proportional response than their senior counterparts. Most responses were second year students (n = 164 students, 37.7%), followed by first year (n = 152 students, 34.9%), fifth year (n = 44 students, 10.1%), third year (n = 38 students, 8.7%), and fourth year (n = 37 students, 8.5%). Most of the participants (59.3%) had a GPA of 4.5–5.0 (Table 1).

**Table 1:** Demographics and characteristics of medical students (n = 435).

Variable		
Age, years	Mean ± SD	20.15 ± 1.73
	Range	17–27
Gender	Male	246 (56.6%)
	Female	189 (43.4%)
Nationality	Saudi	314 (72.2%)
	Non-Saudi	121 (27.8%)
University	KSAU-HS (Riyadh)	79 (18.2%)
	KSAU-HS (Jeddah)	89 (20.5%)
	Alfaisal University (Riyadh)	174 (40.0%)
	King Abdulaziz University (Jeddah)	93 (21.4%)
Current academic year	First year	152 (34.9%)
	Second year	164 (37.7%)
	Third year	38 (8.7%)
	Fourth year	37 (8.5%)
	Fifth year	44 (10.1%)
GPA	4.5–5.0	258 (59.3%)
	4.0–4.4	67 (15.4%)
	3.5–3.9	61 (14.0%)
	3.0–3.4	22 (5.1%)
	2.5–2.9	19 (4.4%)
	Missing	8 (1.8%)

Data are given as n (%) unless otherwise noted.

Table 2 presents information about the various associations between the involvement in research and participants’ characteristics, various significant links have been established. The table mentions the significance of such associations and their respective confidence intervals (CI) with the lower (LB) and upper bounds (UB). First when looking at which university the participants attended, the study revealed that the highest research involvement was from participants in Al Faisal (69/435) followed by KSAUHS-Jeddah (56/435) with a significance p value of 0.001 meaning the relationship between involvement in research and university is a significant relationship. Another factor was the GPA, showing that students with higher GPAs (4.5–5.0) produce more research (134/435) with a significance p value of 0.016. Hence, indicating a relationship between higher GPAs and output of research. Finally academic year, our study presented that second-year students were more likely to participate in research when compared to other years which revealed to be statistically significant.

**Table 2:** Involvement in research vs participants’ characteristics.

Variable	Involvement in research	Yes	No	Significance	CI	
					LB	UB
Nationality	Saudi	154 (35.4%)	160 (36.8%)	0.327	-	-
	Non-Saudi	53 (12.2%)	68 (15.6%)			
University	KSAU-HS (Riyadh)	44 (10.1%)	35 (8.0%)	0.001	0.01	0.02
	KSAU-HS (Jeddah)	56 (12.9%)	33 (7.6%)			
	Alfaisal University (Riyadh)	69 (15.9%)	105 (24.1%)			
	King Abdulaziz University (Jeddah)	38 (8.7%)	55 (12.6%)			
Current academic year	First year	34 (7.8%)	118 (27.1%)	0.000	0.000	0.000
	Second year	71 (16.3%)	93 (21.4%)			
	Third year	25 (5.7%)	13 (3.0%)			
	Fourth year	34 (7.8%)	3 (0.7%)			
	Fifth year	43 (9.9%)	1 (0.2%)			
GPA	4.5–5.0	134 (30.8%)	124 (28.5%)	0.019	0.016	0.021
	4.0–4.4	35 (8.0%)	32 (7.4%)			
	3.5–3.9	20 (4.6%)	41 (9.4%)			
	3.0–3.4	8 (1.8%)	14 (3.2%)			
	2.5–2.9	5 (1.1%)	14 (3.2%)			
	Missing	5 (1.1%)	3 (0.7%)			

Although there are many motivating factors, the top three strongly agreed upon factors were “admission into residency” (56.8%), “interest in research” (41.8%), and “aid in clinical decision making” (31%). The strongly disagreed upon motivators were “financial return” (11.7%), “competition between students” (10.3%), and “elective research programs” (3.2%). When looking at barriers, 51.5% of students identified “lack of time” as being the strongest barrier, followed by “lack of mentoring” (41.8%) and “lack of knowledge” (34%). When looking at the constraints with the lowest effect, they were primarily “lack of financial return” (12.6%), “limited database access/data” (5.7%), and “irrelevancy to preferred specialty” (5.3%) (see Appendix 2 in S1 Appendix).

**Fig 1:**
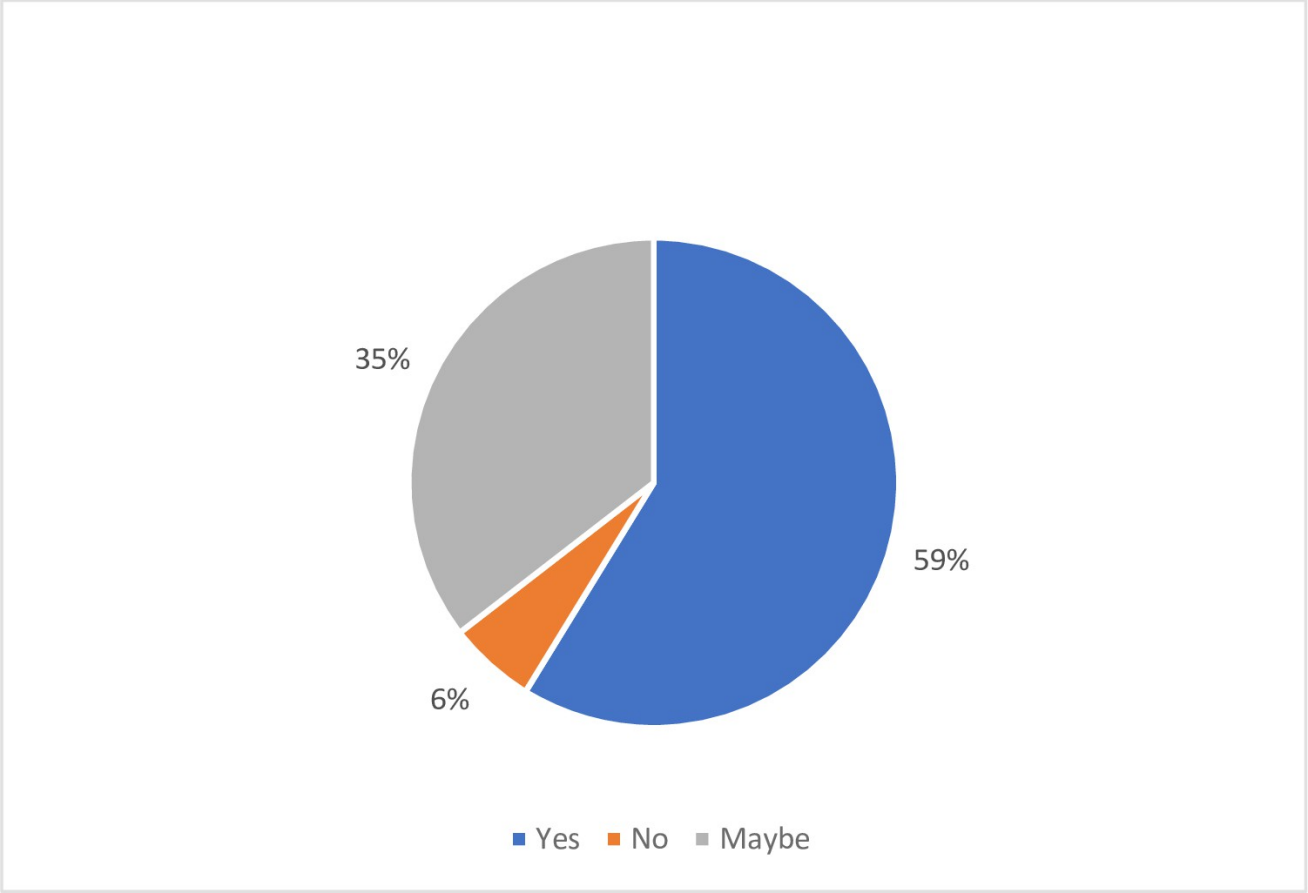
Students’ opinion on the significance of research to their career.

Most of the students thought that research is an important aspect of their future careers (59%). 35% of the participants chose the “maybe” variable, which could be explained by either the students do not know whether it affects their career or simply they do not have any interest in research but are aware of its effect on their future careers. “No” is the “not critical” variable. Students that chose “no” (6%) did not consider research a significant aspect of their future careers (Fig 1).

**Fig 2:**
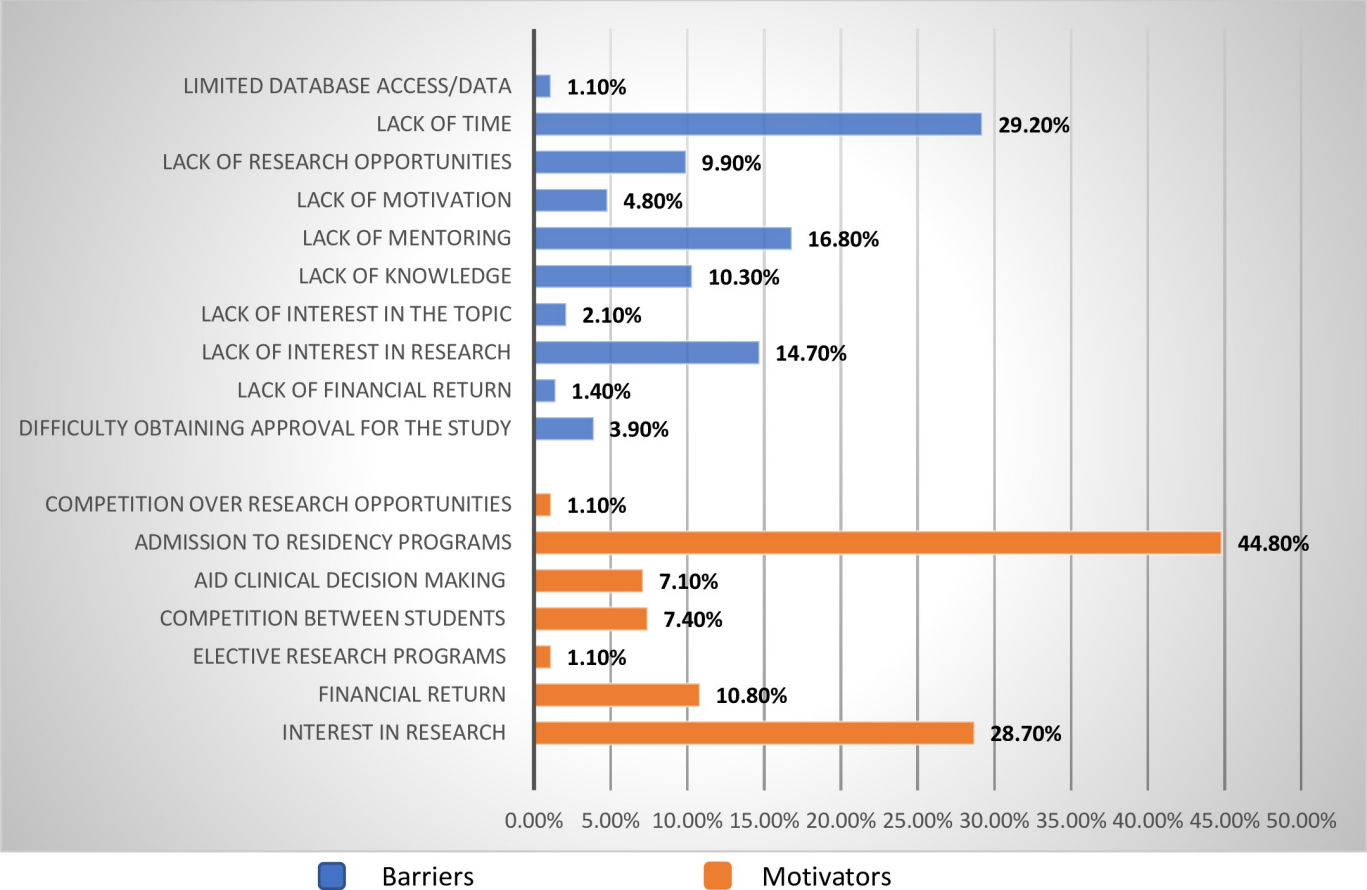
Reported factors that influence the conduct of research.

Fig 2 presents the factors with the greatest influence on students when thinking about undergraduate research. The blue color represents the barriers, while the orange color represents the motivators. “Admission to residency” was selected as having the greatest motivating influence on students (44.8%), followed by “interest in research” (28.7%) and “financial return” (10.8%). The other factors had <10% influential effect on research. In contrast, the variables with the least motivating influence were “financial return” (34.7%), “competition between students” (28.3%), and “elective research programs” (17.5%). The barriers with the greatest influence were “lack of time” (29.2%), “lack of monitoring” (16.8%), and “lack of interest in research” (14.7%), but “lack of interest in research” was within the top three of greatest barriers even though the majority of participants selected it as the least influential barrier (32.9% vs. <10% for others) (see Appendix 4 in S1 Appendix for more information).

Regarding factors that may have influenced how the participants answered the survey questions. When looking at previous research experiences, current research involvement, and relevance to future residency. A total of 207 (47.6%) students were involved in research projects, whereas 228 (52.4%) were not involved in research. A total of 191 (43.9%) students selected the factors presented in Fig 2 based on previous research experience. Most of the participants (93.8%) were interested in participating in research projects. From those who participated in research, 130 (29.9%) students are in research projects that are not related to their desired specialties. Most of them have been provided with research articles (27.6%) (see Appendix 3 in S1 Appendix).

**Fig 3:**
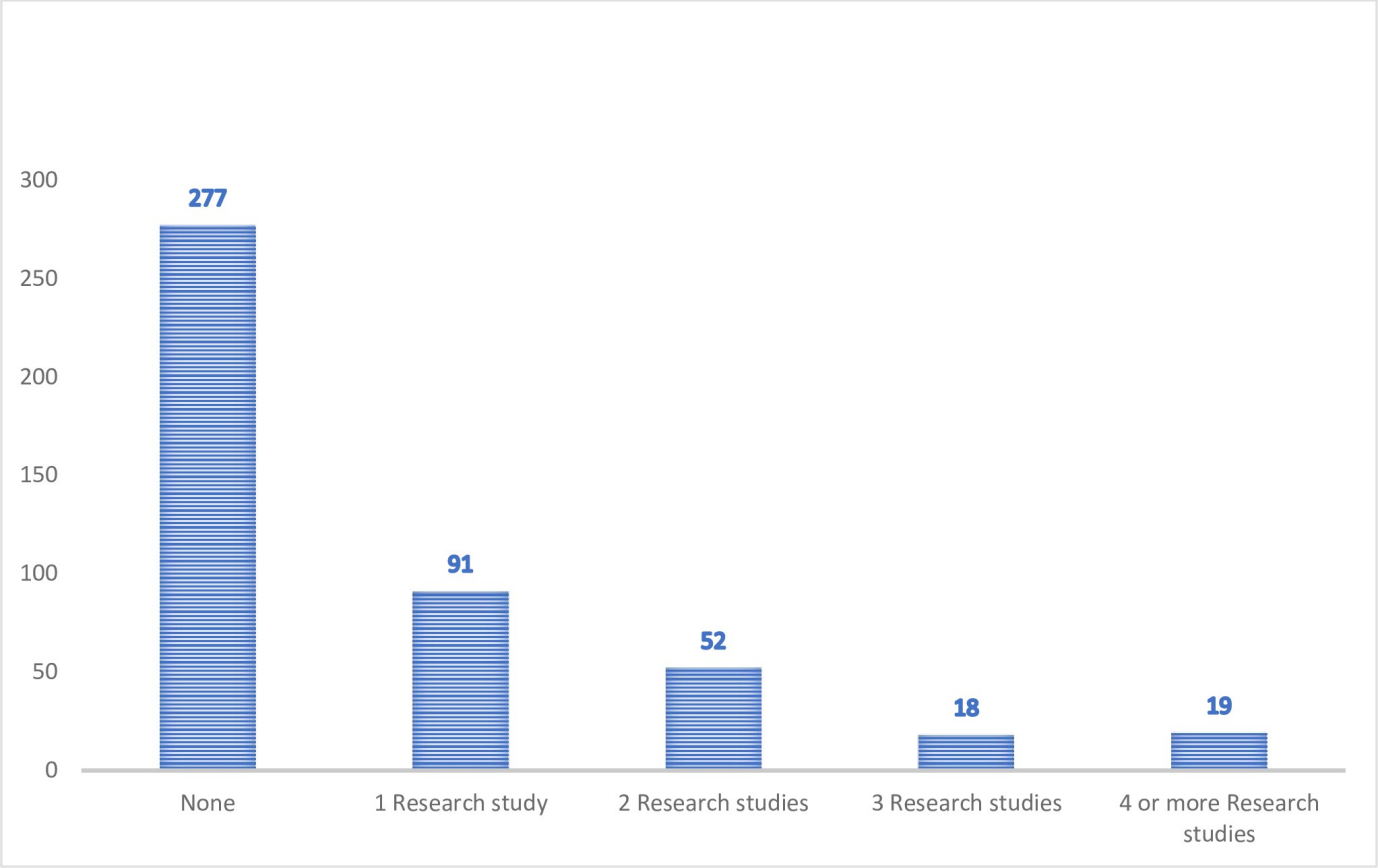
Number of students that have participated in research studies.

Fig 3 presents the number of research studies the participants were involved in. A high proportion of them were not involved in research projects at the time (277/435). While 91/435 participants were involved in 1, 52/435 were involved in 2, 18/345 were involved in 3, and 19/435 were involved in 4 or more research studies.

**Fig 4:**
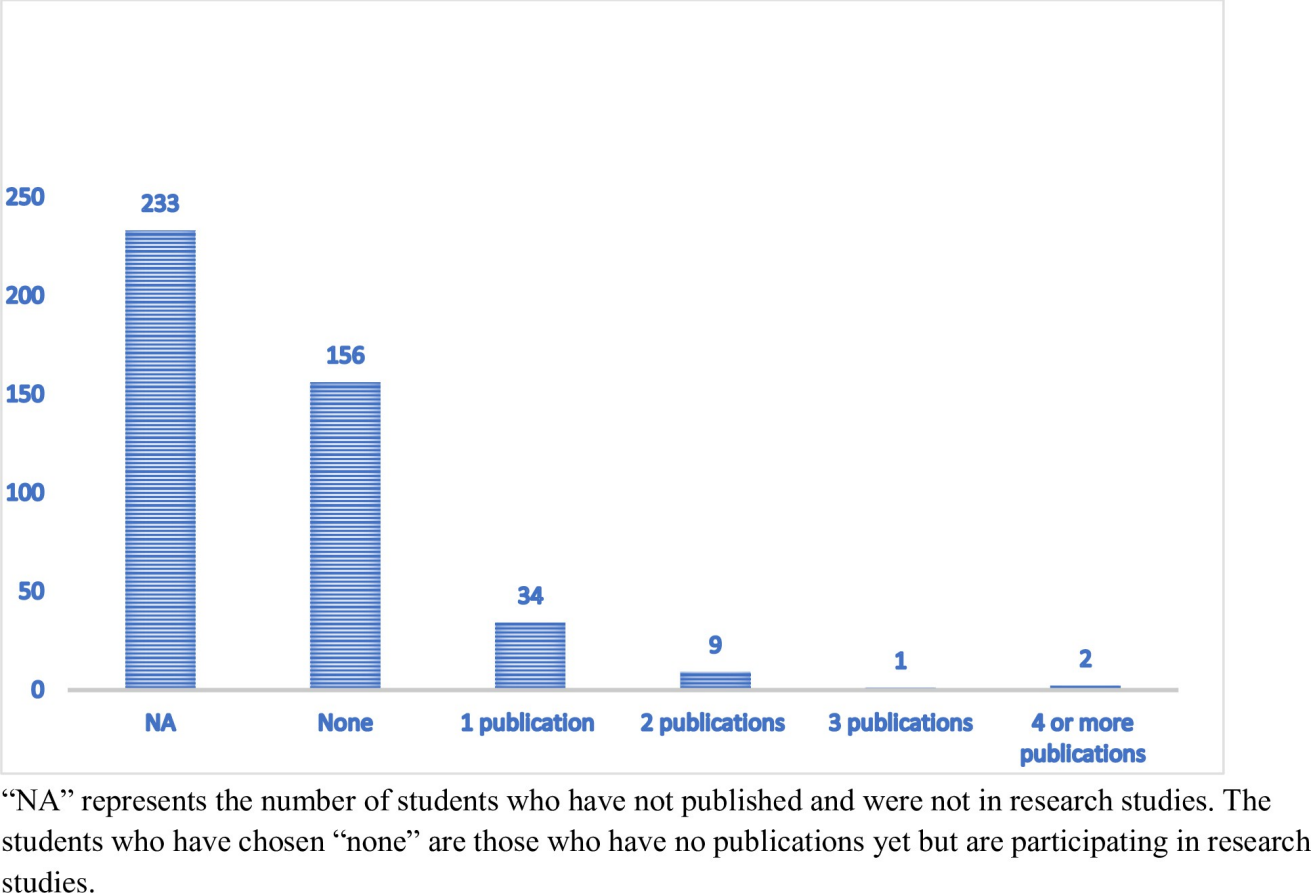
Number of publications by participants.

Fig 4 illustrates the number of publications per participant. The number of participants decrease as the number of publications increase. Students that chose the “NA” variable (232/435) did not publish any research article and were not involved in any research studies. The “none” variable explains those who have no publications yet but are participating in research studies (156/435).

## Discussion

To our knowledge, our study is the first multicenter study in the Middle east with a large sample size to examine the motivators and barriers medical students face when pursuing research. The major findings included: first, less than half of our study’s participants were involved in research (47.6%). Second, the participants identified a lack of time, a lack of mentoring, and a lack of interest in research as the top three barriers influencing research conduct. Third, our study also revealed three fundamental motivators: admission to residency programs, interest in research, and the financial return.

When looking at involvement of medical students in research, our study revealed that only 47.6% involvement in research. While the range might be variable worldwide, nevertheless, universities have various methods promoting research. A study done in Stanford University, revealed that 90% of medical students were participating in research [6,8]. This was mainly attributed to the curricular system, which pushed them to ask questions, review the literature cautiously, and analyze the data. Furthermore, participants in that study reported to be motivated to pursue research due to that experience [8]. Another study in New Zealand, presented a 38.3% involvement of their participants in research which was surprisingly lower than our studies percentage [9]. A study on 13 medical programs conducted in six Brazilian states revealed that only 60% of students participated in research. The study stated that the main barrier that students faced was lack of institutional incentive which also corresponds to our study [10]. Many medical schools do not offer research education programs which leaves students lost, confused, and overwhelmed when it comes to research. Another study in Ayub Medical College (Pakistan) found that 59.5% of medical students were involved in research, the study states that the main motivator to conduct research was learning research methodology [11]. Mainly students here viewed conducting research as a way to acquire more skills for their future careers, they viewed it in a hands-on approach, the more research they participated in the more skills they could pick up. This approach was not encountered in our study as students preferred to have a basic skill set before conducting research.

Our study’s major findings were aligned with those of a different study conducted at an Egyptian medical school [2]. A lack of time was identified as the most significant barrier to clinical research participation in multiple studies [1,3,4,12–14]. Although research participation is essential for medical students to enhance their knowledge and ensuring continuous development and discoveries in all fields, these students seem to fall behind due to lack of time. Such students are busy shuffling various heavy subjects and working full hours to get their medical degree. Nevertheless, the lack of awareness and guidance plays a critical role in preventing medical students from conducting research [15]. Compulsory curriculum methods have been used in various intitutes to promote research. Students are exposed early on to research activities, so teaching about research methodology and elective research programs would assist young students that are willing to pursue research [7]. Various contributions encourage students to participate in research, develop better research skills, and ultimately pursue an academic medical path or career [7]. A study involving Pakistani medical students indicated that teaching could enhance their understanding of health research [13]. According to their research, attendance at a course on research methodology has a beneficial short-term influence on students’ attitudes toward science. They believed that this favorable effect should be sustained by vertically integrating training into the core education [14]. Another study done at Stellenbosch University suggested that embedding research courses into the curriculum could face many obstacles. The barriers limiting the incorporation of research studies in the curriculum include lack of infrastructure and supervisory support, lack of time and space in the curriculum itself, and lack of research opportunities, which could lead to misperceptions about research or lack of competence and self-efficacy. Nonetheless, it is evident that some obstacles, such as time limits, are universal and activities such as research, which are not a priority for undergraduate students, tend to be sidelined [16]. Lack of mentoring was reported in different studies as the main factor limiting students from conducting research. When a mentor guides an individual through the learning process, acquiring new abilities is simple. Mentors not only help with skill development, but also evaluate progress and provide corrective advice. Other studies reached similar conclusions regarding the significance of research mentoring for medical students [1,2,4]. Research participation is influenced by the attitudes and perceptions held by the students towards research; therefore, it is important to understand and document them. Our study also revealed a distinctive third barrier, which was lack of interest in research. Unlike various previous studies, 64/435 (14.7%) participants expressed their lack of interest in pursuing research or research-related programs.

Admission to residency programs and interest in research were found to be the most influential factors in conducting research. This aligns with various previous studies, one of which was done among Alfaisal medical students, where three key motivators were identified: the belief that research helps with acceptance into competitive residency programs, the belief that research helps students stand out when writing their curriculum vitae and having the chance to publish in a peer-reviewed journal [17]. Additionally, Rosenkranz et al. explored a cohort of 579 students and identified multiple motivators. First, previous research experience was identified as one of the main motivators [18]. Second, educational advancements as in clinical year or senior students were more likely to participate. A third motivator was the belief that research will improve future chances of success in a medical career [18]. Contrary to our expectations financial return was revealed as one of the main motivators to research participation. This finding did not align with other studies.

Research participation is an important factor that correlates with many of the variables we included in this study. Students with higher GPA rates revealed to be more involved in research when compared to their counterparts. However, it is important to mention that a high proportion (59.3%) of participants in the study were from the higher GPA rates (4.5–5.0). Which could be explained by two main reasons; higher achieving students have a better grasp of the curriculum and thus want to explore it further via research. Second, higher achieving students have better relationships with doctors, putting them at the forefront when it comes to new research opportunities. Moreover, a significance between different genders and research participation arose. Saudi students revealed to be more involved in research studies than non-Saudis (34.5% vs 12.2%), however, it was proven to be not statistically significant. A correlation between the academic years and research participation was also obtained. This revealed a higher research involvement in second year students (16.3%), fifth year students (9.9%), followed by first and fourth-year students (8.8%). Nevertheless, this can be explained by the higher number of participants being from first and second year compared to senior years.

Our study had several strengths. It included four large universities in Saudi Arabia (private and public), being the largest sample size and first of its kind in the Middle East and enrolled a random snapshot of students’ perspective from various academic levels. However, the study had some limitations including inherent bias that are prone to happen in cross-sectional surveys, i.e.: students with a higher GPA were more likely to participate in surveys, and hence overestimating the proportion of medical students’ involvement in research. Furthermore, our study results could not be generalized to all universities in Saudi Arabia since most of them were not involved in the study.

## Conclusions

The most influential barrier in conducting research was lack of time. Additionally, admission into residency programs was revealed to be the most influential motivator, rather than genuine interest in research. Our study is a call for action to raise awareness among medical students about the importance of research and to provide solutions to overcome these barriers. Future studies are needed to include larger number of universities and medical students to ensure validity of our study results.

## Supporting information

S1 Appendix (DOCX)

S1 File (ZIP)
