# Correction: Motivators and barriers to research participation among medical students in Saudi Arabia

**DOI:** 10.1371/journal.pone.0306375

**Published:** 2024-06-27

**Authors:** Rakan K. Alhabib, Noara Alhusseini, Anas G. Aboalsamh, Ghaith Adi, Aya Ismail, Amro Hajja, Duaa Alammari, Ziad Khalil, Maha A. Alharbi, Sarah K. Albahiti

There is an error in affiliation 4 for author Duaa Alammari. The correct affiliation 4 is: College of Public Health and Health Informatics, King Saud bin Abdulaziz University for Health Sciences (KSAU-HS), King Abdullah International Medical Research Center, Riyadh, Saudi Arabia.
