# Correction: Motivators and barriers to research participation among medical students in Saudi Arabia

**DOI:** 10.1371/journal.pone.0321127

**Published:** 2025-03-25

**Authors:** Rakan K. Alhabib, Noara Alhusseini, Anas G. Aboalsamh, Ghaith Adi, Aya Ismail, Amro Hajja, Duaa Alammari, Ziad Khalil, Maha A. Alharbi, Sarah K. Albahiti

There is an error in affiliation 6 for author Sarah K. Albahiti. The correct affiliation 6 is: Radiology

Department, College of Medicine, King Abdulaziz University, Jeddah, Saudi Arabia.
