# Supplementary material for: Motivators and barriers to research participation among medical students in Saudi Arabia
Source: PLoS One. 2023 Apr 27;18(4):e0284990. doi: 10.1371/journal.pone.0284990 (PMC10138799; doi:10.1371/journal.pone.0284990)
Supplement: S1 Appendix — (DOCX) [file pone.0284990.s001.docx]

# Appendices

## Appendix 1

**The questionnaire:** The distributed questionnaire was developed based on assessing various previously published questionnaire’s [5,7]. The survey was distributed through various social media platforms. It included general questions about respondents and questions related to research motivators and barriers. All data will be accessible to only the authors. Students participating in the study will be volunteering and consent will be obtained from the students to participate in the study. Approval from the Institutional Review Board of all universities will be sought before data collection. We will distribute our survey through Google forms.

**Survey in English**

**Greetings,**

**We are inviting you to participate in our study. We are a research team from King Saud Bn AbdulAziz University for Health Sciences, the College of Medicine at Alfaisal University, and King Abdulaziz University in Jeddah. The main objective of this study is to explore the barriers and motivators influencing medical students to participate in research in Riyadh, Saudi Arabia. The survey contains a series of statements about your experiences and activities relevant to your personal career. It asks you to select the response that most closely reflects your personal experience or opinion. Your feedback is appreciated and extremely valued. Participation is voluntary, and you may withdraw at any time. All answers will be kept confidential. Filling out the survey will be construed as consent. Filling out the survey takes from 2 to 5 minutes.**

*** Participation is restricted to medical students only.**

**Contact Information: Aya Ismail**

**Department: College of Medicine, Alfaisal University**

**Email: Aeismail@alfaisal.edu**

1. **Are you a medical student? (if they answer no, it ends the survey)**

○ **Yes**

○ **No**

***[If participants answer no, the survey will take them to a section saying: “Thank you for your time. Unfortunately, our research is limited only to medical students. Please click on "submit" before exiting.” If participants answer yes, they will continue with the survey questions.]***

1. **What is your age? Drop down menu**
2. **What is your nationality?**

○ **Saudi**

○ **Non-Saudi**

1. **Gender**

○ **Male**

○ **Female**

1. **What university do you study at?**

○ **Alfaisal University**

○ **King AbdulAziz University**

○ **King Abdulaziz University of Health Sciences**

○ **Other : please specify**

1. **What year are you currently in?**

○  **First year**

○  **Second year**

○  **Third year**

○ **Fourth year**

○ **Fifth year**

1. **What is your current GPA? (out of 4)**

○ **2.0 - 2.4**

○ **2.5 - 2.9**

○ **3.0 - 3.4**

○ **3.5 - 3.74**

○ **3.75 - 4.0**

○ **It's out of 5**

1. **If it's out of 5, please choose from the following?**

○ **2.5 - 2.9**

○ **3.0 - 3.4**

○ **3.4 - 3.9**

○ **4.0 - 4.4**

○ **4.5 - 5.0**

1. **Have you been involved in research before?**

○ **Yes**

○ **No**

1. **Are you currently working on a research project?**

○ **Yes**

○ **No**

1. **Are you willing to participate in a research project in the future?**

○ Yes

○ No

1. **Please select the degree to which the stated factors play a role/motivate you for conducting undergraduate medical research:**

|  | **Strongly Agree** | **Agree** | **Neutral** | **Disagree** | **Strongly Disagree** |
| --- | --- | --- | --- | --- | --- |
| **Interest in research as a career** |  |  |  |  |  |
| **Financial return** |  |  |  |  |  |
| **Competition between students** |  |  |  |  |  |
| **Admission into residency programs** |  |  |  |  |  |
| **Aid clinical decision making** |  |  |  |  |  |
| **Elective research programs** |  |  |  |  |  |

1. **Of the factors you picked in the previous question, which would you say has the MOST influence?**

○ **Drop down menu with the same factors as question**

1. **Of the factors in the previous question, which would you say has the LEAST influence?**

○ **Drop down menu with the same factors as question**

1. **If you have another motivating factor/s that are not listed, please write it down?**

○  **Answer should be limited to 15 words**

1. **In your personal opinion, which of the following barriers prevent medical students from participating in undergraduate research?**

|  | **Strongly Agree** | **Agree** | **Neutral** | **Disagree** | **Strongly Disagree** |
| --- | --- | --- | --- | --- | --- |
| **Lack of interest in research** |  |  |  |  |  |
| **Research is not relevant to my preferred specialty** |  |  |  |  |  |
| **Lack of knowledge** |  |  |  |  |  |
| **Lack of research opportunities** |  |  |  |  |  |
| **Lack of time** |  |  |  |  |  |
| **Lack of mentoring** |  |  |  |  |  |
| **Financial issues** |  |  |  |  |  |
| **Limited database access/data** |  |  |  |  |  |
| **Lack of interest in the topic** |  |  |  |  |  |
| **Lack of motivation** |  |  |  |  |  |
| **Competition over research opportunities** |  |  |  |  |  |
| **Difficulty obtaining approval for the study** |  |  |  |  |  |

1. **Of the factors stated previously, which would you say has the MOST influence?**

○ **Drop down menu with the same factors as question**

1. **Of the factors you picked in the previous question, which would you say has the LEAST influence?**

○ **Drop down menu with the same factors as question**

1. **If you have other barrier/s that are not listed, please write it down?**

○  **Must be limited to 15 words**

1. **Are these motivators and barriers based on your previous research experience?**

● **Yes or No**

● **If yes, how many research studies have you participated in?**

○ **1 research study**

○ **2 research studies**

○ **3 research studies**

○ **4 or more research studies**

● **How many articles have you published?**

○ **None**

○ **1 publication**

○ **2 publications**

○ **3 publications**

○ **4 or more publications**

1. **Is research important for your career?**

○ **Yes**

○ **Partially**

○ **No**

1. **If yes, why is it important?**
2. **If not, why is it not important?**

## Appendix 2

**Reported factors that play a role in conducting research**

|  | **Strongly Agree** | **Agree** | **Neutral** | **Disagree** | **Strongly Disagree** |
| --- | --- | --- | --- | --- | --- |
| **Interest in Research** | 182 (41.8%) | 134 (30.8%) | 93 (21.4%) | 15 (3.4) | 11 (2.5%) |
| **Financial Return** | 87 (20%) | 135 (31%) | 110 (25.3%) | 52 (12%) | 51 (11.7%) |
| **Competition between Students** | 98 (22.5) | 122 (28%) | 110 (25.3%) | 60 (13.8%) | 45 (10.3%) |
| **Admission to Residency Programs** | 247 (56.8%) | 136 (31.3%) | 44 (10.1%) | 6 (1.4%) | 2 (0.5%) |
| **Aid in Clinical Decision Making** | 137 (31.5) | 165 (37.9%) | 99 (22.8%) | 24 (5.5%) | 10 (2.3%) |
| **Elective Research Programs** | 103 (23.7%) | 127 (29.2%) | 160 (36.8%) | 31 (7.1%) | 14 (3.2%) |
| **Lack of Interest in Research** | 111 (25.5%) | 171(39.3%) | 79 (18.2%) | 82 (12%) | 22 (5.1%) |
| **Irrelevancy to Preferred Specialty** | 61 (14%) | 172 (39.5%) | 97 (22.3%) | 82 (18.9%) | 83 (5.3%) |
| **Lack of Mentoring** | 182 (41.8%) | 142 (32.6%) | 83 (19.1%) | 24 (5.5%) | 4 (0.9%) |
| **Lack of Time** | 224 (51.5%) | 138 (31.7%) | 46 (10.6%) | 26 (6 %) | 1 (0.2%) |
| **Lack of Knowledge** | 148 (34%) | 164 (37.7%) | 76 (17.5%) | 42 (9.7%) | 5 (1.1%) |
| **Lack of Research Opportunities** | 125 (28.7%) | 135 (31 %) | 111 (25.5%) | 54 (12.4%) | 10 (2.3%) |
| **Lack of Financial Return** | 53 (12.2%) | 83 (19.1%) | 148 (34%) | 96 (22.1%) | 55 (12.6%) |
| **Limited Database Access/Data** | 67 (15.4%) | 117 (26.9%) | 144 (33.1%) | 82 (18.9%) | 25(5.7%) |
| **Lack of Interest in the Topic** | 87 (20 %) | 180 (41.1%) | 104 (23.9%) | 53 (12.2%) | 11(2.5%) |
| **Lack of Motivation** | 132 (30.3%) | 165 (37.9%) | 74 (17 %) | 52(12.8%) | 12 (2.8%) |
| **Competition over Research Opportunities** | 86 (19.8%) | 130(29.9%) | 131(30.1%) | 70(16.1%) | 18 (4.1%) |
| **Difficulty Obtaining Approval for the Study** | 88 (20.2%) | 162 (37.2%) | 134(30.8%) | 34 (7.8%) | 17 (3.9%) |

## Appendix 3

**Previous research experiences, current research involvement, and relevance to future residency**

|  | Yes | No | Not applicable |
| --- | --- | --- | --- |
| **Are these motivators & barriers based on your previous research experience?** | 191 (43.9%) | 244 (56.1%) |  |
| **Involvement in research** | 207 (47.6%) | 228 (52.4%) |  |
| **Will to participate in research** | 408 (93.8%) | 27 (6.2%) |  |
| **Have you been provided any research articles** | 120 (27.6%) | 71 (16.3%) | 244 (56.1%) |

## Appendix 4

**Reported factors that influence the conduct of research**

|  | **Greatest Influence** | **Least Influence** |
| --- | --- | --- |
| **Interest in Research** | 125 (28.7%) | 49 (11.3%) |
| **Financial Return** | 47 (10.8%) | 151 (34.7%) |
| **Elective Research Programs** | 5 (1.1%) | 76 (17.5%) |
| **Competition Between Students** | 32 (7.4%) | 123 (28.3%) |
| **Aid in Clinical Decision Making** | 31 (7.1%) | 33 (7.6%) |
| **Admission to Residency Programs** | 195 (44.8%) | 3 (0.7%) |
| **Competition over Research Opportunities** | 5 (1.1%) | 5 (1.1%) |
| **Difficulty Obtaining Approval for the Study** | 17 (3.9%) | 35 (8%) |
| **Lack of Financial Return** | 6 (1.4%) | 23 (5.3%) |
| **Lack of Interest in Research** | 64 (14.7%) | 143 (32.9%) |
| **Lack of Interest in the Topic** | 9 (2.1%) | 34 (7.8%) |
| **Lack of Knowledge** | 45 (10.3%) | 11 (2.5%) |
| **Lack of Mentoring** | 73 (16.8%) | 25 (5.7%) |
| **Lack of Motivation** | 21 (4.8%) | 20 (4.6%) |
| **Lack of Research Opportunities** | 43 (9.9%) | 14 (3.2%) |
| **Lack of Time** | 127 (29.2%) | 16 (3.7%) |
| **Limited Database Access/Data** | 5 (1.1%) | 21 (4.8%) |
